# Males, but not females, perform strategic mate searching movements between host plants in a leaf beetle with scramble competition polygyny

**DOI:** 10.1002/ece3.4121

**Published:** 2018-05-07

**Authors:** Danilo G. Muniz, Martha L. Baena, Rogelio Macías‐Ordóñez, Glauco Machado

**Affiliations:** ^1^ Programa de Pós‐Graduação em Ecologia Departamento de Ecologia Instituto de Biociências Universidade de São Paulo São Paulo Brazil; ^2^ LAGE do Departamento de Ecologia Instituto de Biociências Universidade de São Paulo São Paulo Brazil; ^3^ Instituto de Investigaciones Biológicas Región Xalapa Universidad Veracruzana Xalapa Mexico; ^4^ Red de Biología Evolutiva Instituto de Ecología A.C. Xalapa Xalapa Mexico

**Keywords:** Chrysomelidae, mate searching strategy, mating system, mobility, scramble competition, sexual harassment, sexual selection, *Solanum*

## Abstract

Mate searching is assumed to be performed mostly by males, but when females benefit from multiple mating or are under risk of failing to mate, they may also perform mate searching. This is especially important in scramble competition polygynies, in which mate searching is the main mechanism of mate competition. Typically, more mobile individuals are expected to achieve higher mating success because mobility increases their probability of finding mates. If we assume individual movements are mainly explained by mate searching in scramble competition polygynies, we can investigate searching strategies by asking *when* individuals should leave their location and *where* they should go. We hypothesize that individuals will leave their locations when mating opportunities are scarce and will seek spatially close sites with better mating opportunities. We tested these hypotheses for males and females of *Leptinotarsa undecimlineata*, a leaf beetle with scramble competition polygyny in which both sexes are promiscuous. Individuals mate and feed exclusively on *Solanum* plants, and thus, individual movements can be described as switches between plants. Females were less likely than males to leave isolated plants, and both males and females moved preferentially to neighboring plants. Males were more likely to leave when the local number of females was low, and the number of males was high. They moved to plants with more females, a behavior consistent with a mate searching strategy. Females were more likely to move to plants with fewer males and many females, a behavior consistent with male harassment avoidance. Strategic movement is widely considered in foraging context, but seldom in a mate searching context. Considering that selection to minimize searching costs, maximize mating success, and minimize harassment may be ubiquitous in nature, we argue that strategic movements by mate searching individuals are likely to occur in many species.

## 1 INTRODUCTION

It is often assumed that males perform most, or all, of the mate searching because they would benefit from multiple mating, whereas females would rarely need more than one copulation to fertilize all their eggs (Andersson, 1994). However, recent empirical evidence shows that females of many species benefit from multiple mating (Kvarnemo & Simmons, 2013; Parker & Birkhead, 2013) or may face the risk of failing to mate (Rhainds, 2010). Under these two conditions, we would expect females also to benefit from investing in mate searching. Accordingly, theoretical models predict that although males should invest more in mate searching than females, females should also perform some investment in mate searching (Fromhage, Jennions, & Kokko, 2016). Thus, empirical and theoretical findings suggest that mate searching should be studied both in males and females, and that hypotheses about mate searching should be tested for both sexes (Kokko, Klug, & Jennions, 2013).

Mate searching is especially important in scramble competition polygynies (SCPs), in which the main mechanism of competition for mates is mate searching (Andersson, 1994). Hence, in SCPs, we can expect that more mobile males attain higher mating success (Herberstein, Painting, & Holwell, 2017). Indeed, a positive relationship between male movement and mating frequency has been reported for some species of reptiles (Madsen, Shine, Loman, & Håkansson, 1993), mammals (Sandell, 1986; Stockley, Searle, Macdonald, & Jones, 1994), birds (Oh & Badyaev, 2010), and insects (Alcock, 1980). However, contrary to that expectation, negative relationships between male movement and mating success have been reported for a few species (Baena & Macías‐Ordóñez, 2015; Brown & Weatherhead, 1999). Regardless of the relationship between individual movement and mating success, most of the studies focus only on males, ignoring the potential role of female movement in mate searching. If selection to increase mating encounters and hence mating success is assumed, one would expect that more mobile females would have higher copulation success, similarly to males. Currently, though, data on female movement and copulation rate are rare and both positive (Rank, Yturralde, & Dahlhoff, 2006) and negative relationships have been reported (Baena & Macías‐Ordóñez, 2015).

Negative relationships between movement and mating success for both males and females remain largely unexplained. However, Baena and Macías‐Ordóñez (2015) recently proposed a still untested mechanistic hypothesis according to which individuals attaining high mating success should remain where they are, whereas individuals unable to copulate should move in search of more favorable mating conditions (i.e., places with more potential mates or less competition for the available mates). This hypothesis reverses the commonly assumed cause and effect relationship between mating and movement. According to this rationale, increased mobility does not necessarily lead to more copulations in SCPs. Rather, increased mobility can be the result of low copulation frequency, which could explain the negative correlation between movement and mating success found in some species (Baena & Macías‐Ordóñez, 2015) or populations of the same species (Brown & Weatherhead, 1999).

If individual movement in SCPs is mostly explained by mate searching, two questions immediately arise: (1) *When to move?* and (2) *Where to go?* When mating sites are discrete patches, such as water pools used by many frogs or host plants used by phytophagous insects, individuals should prefer short movements, which require less energy and expose individuals to less predation risk (Kasumovic, Bruce, Herberstein, & Andrade, 2006; Polis, Barnes, & Seely, 1998). Whenever movements are made between sites that are embedded in a space with potential hazards to the moving individuals, the spatial structure of the environment should play a role in both *when* and *where* movement decisions. Thus, individuals should be more likely to move when local conditions are unfavorable and when there are alternative sites available nearby. The prediction is that individuals in central mating sites, spatially close to alternative sites, should be more likely to move than individuals in isolated sites, and they should move preferentially to nearby sites. The social environment may also be important, so that individual movement should be influenced by the number of potential competitors (i.e., same‐sex individuals) and mating partners (Holwell, Allen, Goudie, Duckett, & Painting, 2016). The prediction is that individuals should be more likely to leave sites with many competitors and few potential mates. Moreover, once individuals have left a site, the prediction is that they should prefer to move toward sites with fewer competitors and more potential mates.

Here, we studied the movements of the phytophagous leaf beetle *Leptinotarsa undecimlineata* (Chrysomelidae) to test the predictions presented above. Individuals of this species live most of their adult lives on host plants of the family Solanaceae (Baena & Macías‐Ordóñez, 2012), and thus, their movements can be described as switches between individual host plants. This is a species with SCP in which mobility is negatively correlated with mating frequency for both males and females (Baena & Macías‐Ordóñez, 2015). Males defend females only during copulation and immediately after it, so that their main mating tactic is to search for females on host plants (Baena & Macías‐Ordóñez, 2012). Using detailed daily observations on individual location, copulation frequency, and social environment in each host plant, we tested the following predictions for both males and females: (1) Individuals are more likely to switch between host plants when mating success and availability of mates are low and when the number of competitors is high; (2) this effect should be more pronounced when individuals are in central host plants, that is, plants spatially close to each other; and (3) individuals should move preferentially to nearby host plants and (4) prefer host plants with low number of competitors and high number of potential mates.

## 2 MATERIALS AND METHODS

### 2.1 Study organism

**Figure 1:**
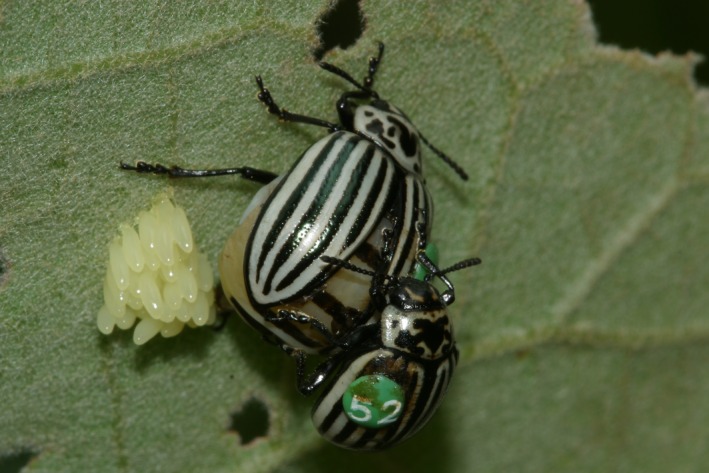
Male and female of the leaf beetle *Leptinotarsa undecimlineata* on the host plant after copulation. Note that the male (below) is smaller than the egg‐laying female and that the abdomen of the female is very inflated due to the egg load in the reproductive tract, so that the two elytra do not touch each other. Photograph by Juan H. García‐Chávez

Larvae and adults of *L. undecimlineata* feed exclusively on two host plant species: *Solanum lanceolatum* and *S. chrysothricum* (Solanaceae). These plants are also used as mating and oviposition sites (Figure 1), so that individuals spend most of the breeding season on the host plants and are rarely found elsewhere. Over the course of the breeding season, both males and females mate repeatedly and promiscuously. Following copulation, males usually perform mate guarding by staying near the female or mounted on her (Figure 1). Males may attempt to displace other males during copulation, but they do not perform prolonged territorial or female defense nor present any visible weapon or sexually dimorphic secondary trait (Baena & Macías‐Ordóñez, 2012, 2015).

### 2.2 Study site and dataset

In this study, we used the dataset of Baena and Macías‐Ordóñez (2012, 2015). Data were collected in a secondary forest replacing an abandoned pasture grassland, close to a fragment of cloud forest at a location known as El Riscal, Central Veracruz, Mexico (19°28ʹ56″N, 96°59ʹ48″W, 1,595 m a.s.l.). Mean monthly temperature in the study area is 20°C (min–max = 12–34°C), and annual precipitation is more than 3,000 mm. The studied population lived on a patch with high density of host plants in an area of about 400 m^2^. Host plants and leaf beetles were individually marked (for more details on the marking procedures and sampling design, see Baena & Macías‐Ordóñez, 2012, 2015). Plants were visited once a day from July 21st to 7th November 2004, which comprises the entire breeding season of *L. undecimlineata*.

In each daily visit, the identity, location (host plant), and mating activities of all individual beetles present in the study area were recorded. Copulations occurred within an 80‐day period, and to test our predictions, we used only the data from this period. Exact spatial coordinates, measured as linear distances from a standardized point, were available for 139 host plants in the study area. We used only information of switches between these spatially located plants. We calculated distances between any two plants using Euclidean distance. We also calculated a measure of spatial centrality for each host plant as the sum of the inverse of the distances to all other plants with known coordinates. Thus, host plants with high centrality were closer to other plants, whereas host plants with low centrality were more spatially isolated.

### 2.3 When to move?

Our predictions are that an individual is more prone to move when: (1) Its mating success is low, (2) mate availability is low, (3) intrasexual competition is high, and (4) when they are in host plants with high centrality. In addition, we predict that (5) the effect of mating success, mate availability, and intensity of intrasexual competition will be more pronounced when individuals are in host plants with high centrality. To test these predictions, we used generalized linear mixed models (GLMMs) with binomial distributions and adopted a *cloglog* link function (Gelman & Hill, 2006; McCullagh, 1984). We built separate models for males and females. In both models, each recapture of a marked individual on the day *D* was a sampling unit. The binary response variable was 0 if the individual was on the same plant and 1 if it had switched plants. The predictor variables were measures taken on the day prior to *D*, that is, *D* − *1*. Therefore, we used an individual's environment and mating success at a given time to predict the individual's behavior at a following time. The predictor variables of the model were as follows: (1) a categorical variable of mating success, which was if the individual was observed mating or not during *D* − *1*; (2) a continuous measure of mate availability, which was the number of individuals of the opposite sex on the same plant on *D* − *1*; (3) a continuous measure of intensity of intrasexual competition, which was the number of individuals of the same sex on the same plant on *D* − *1*; (4) the spatial centrality of the host plant where the individual was found on *D* − *1*; and (5) the interactions between spatial centrality and the other variables.

We centered and standardized all continuous variables before model fitting to produce comparable effect sizes (Schielzeth, 2010). We centered the variables by subtracting the mean value from each observed value and standardized the centered values by dividing each value by the standard deviation of the sample. We included the identities of the beetles and of the host plant where the beetles were on *D* − *1* as random effects in the models. We fitted these models in R 3.4.0 (R Core Team 2017) using the package *lme4* (Bates, Martin, Bolker, & Walker, 2015).

### 2.4 Where to go?

We predict that: (1) individuals should move preferentially to nearby plants, (2) with high number of potential mates, and (3) low number of potential competitors (same‐sex individuals). To test these predictions, we used a multinomial network model (MN model). Although the MN model was originally developed to investigate mate choice, it can be used to investigate choice in other contexts (Muniz, Santos, Guimarães, Nakagawa, & Machado, 2017). We used the MN model because it can accommodate pairwise variables, such as distance between individuals or host plants, and also nonpairwise predictor variables, such as the number of individuals in a given site. Here, we considered each host plant switch as a data point in which an individual chose a host plant to switch to. We assumed that an individual could move to any host plant in the study area, and then, we calculated the probability for each plant switch by an individual based on the predictor variables. We ran separate models for males and females, and in both cases, the predictor variables were as follows: (1) the spatial distance between plants (pairwise), (2) the number of males per plant, and (3) the number of females per plant (both nonpairwise).

According to the MN model, the probability *P*_*ij (h)*_ that an individual *h* at plant *i* goes to plant *j* depends not only on the characteristics of plant *j*, but also on the characteristics of all available host plants in the study area (according to predictions 2 and 3). The equation of the model was:

$$P_{ij(h)}=\frac{\exp\left(G_{j}+A_{h}\times s_{\mathrm{ij}}+B\times M_{j}+C\times F_{j}\right)}{\sum_{k=1}^{N}\exp\left(G_{k}+A_{h}\times s_{\mathrm{ik}}+B\times M_{k}+C\times F_{k}\right)},$$

where *s*_*ij*_ is the distance between plant *i* and plant *j*,* M*_*j*_ is the number of males on plant *j*, and *F*_*j*_ is the number of females on plant *j*, the intercept *G* is a random effect of plant identity, and *N* is the total number of plants in the study area. The model slopes are as follows: *A*, which refers to the effect of spatial distance between plants; *B*, which refers to the effect of the number of males on the plant *j*; and *C*, which refers to the effect of the number of females on the plant *j*. In the fitted model, negative *A* values represent preference for nearby plants, whereas positive values represent preference for faraway plants. Positive *B* values represent preference for plants with many males, whereas negative *B* values represent avoidance of plants with many males. Finally, positive *C* values represent preference for plants with many females, whereas negative *C* values represent avoidance of plants with many females. Therefore, we expected negative *A* values for both males and females, but had different predictions for *B* and *C* values according to the sex of the individual. Given that males should seek plants with few males and many females, we expected *B* to be negative and *C* to be positive. For females, in turn, we expected a positive *B* if they are looking for more male availability and a negative *C* if they are looking for lower female density.

We also included in the models a random effect of individual identity in the distance parameter *A* (following Muniz et al., 2017). Hence, in addition to the model slopes (*A*,* B*, and *C*), the model also had two other parameters: *A*_σ_, which is the standard deviation in *A*_*h*_ values, and *G*_σ_, which is the standard deviation in the intercept *G*. The *G*_σ_ parameter represents variation in host plant attractiveness not explained by any of the model's predictor variables. The *A*_σ_ parameter, in turn, represents individual variation in the response to the distance between host plants. As in the previous analysis, we centered and standardized all predictor variables prior to model fitting.

We fit the models by Markov Chain Monte Carlo (MCMC) adopting a Bayesian approach and using the software R 3.4.0 (R Core Team 2017) and the R package *rstan* (Stan Development Team 2016). For each model, we ran three MCMC chains with 10,000 iterations each, plus 1,000 warm‐up iterations. We adopted Stan's standard uninformative improper priors. For more details on model fitting, please see the tutorial in Muniz et al. (2017).

## 3 RESULTS

### 3.1 When to move?

**Figure 2:**
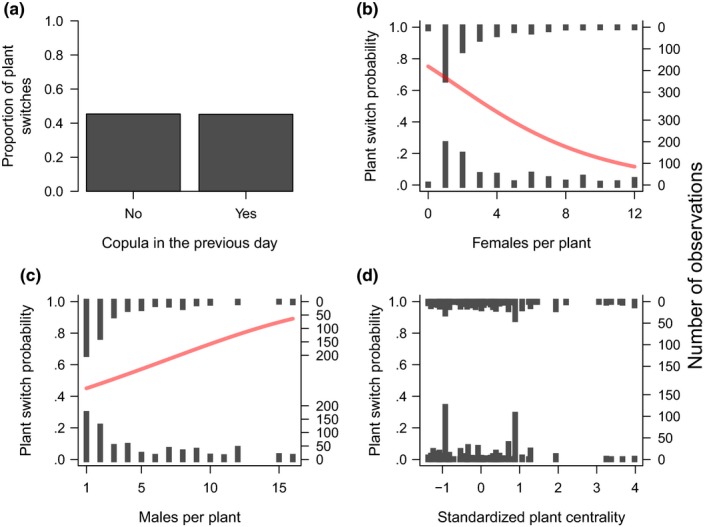
Relationship between plant switches by males of the leaf beetle *Leptinotarsa undecimlineata* and four predictor variables: (a) mating success in the previous day, (b) number of females over the plant, (c) number of males over the plant, and (d) plant spatial centrality. In plots b–d, each bar represents a series of observations with the same value, and bar height represents the number of observations. In plots b–c, redlines represent the probability of plant switch as predicted by the binomial generalized linear mixed model

**Figure 3:**
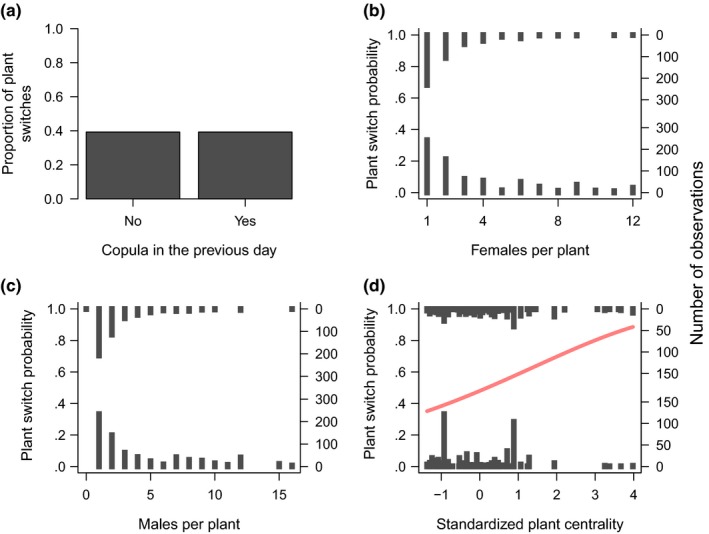
Relationship between plant switches by females of the leaf beetle *Leptinotarsa undecimlineata* and four predictor variables: (a) mating success in the previous day, (b) number of females over the plant, (c) number of males over the plant, and (d) plant spatial centrality. In plots b–d, each bar represents a series of observations with the same value, and bar height represents the number of observations. In plot D, the redline represents the probability of plant switch as predicted by the binomial generalized linear mixed model

Our analysis included a total of 1,093 recaptures from 302 marked males, and 1,141 recaptures from 206 marked females. The proportion of recaptures in which individuals switched plants was 0.45 for males and 0.39 for females. Male movements responded to female availability and to the presence of other males, but not to mating success (Figure 2, Table 1). Males were more likely to switch plants when female availability was low (Figure 2b) and when there were many males on the same plant they were (Figure 2c). Males did not respond to plant spatial position (Table 1), but we observed a marginally nonsignificant positive interaction between spatial centrality and the number of females per plant (Table 1). This interaction can be interpreted as males being less sensitive to local female availability when they are on centrally located plants. Female movement, in turn, responded only to the spatial position of plants. They were more likely to switch plants when in central plants (Figure 3d, Table 1), and their movement probability was not influenced by mating success, by the number of males, or the number of females per plant (Figure 3a–c, Table 1).

**Table 1:** Summary of the generalized linear mixed models that investigate *when* males and females of the leaf beetle *Leptinotarsa undecimlineata* should switch between host plants

Predictor variable	Estimate	z‐value	*p*‐value
*Females*			
(Intercept)	−0.43 ± 0.13	−3.17	**<.01**
Spatial centrality	0.3 ± 0.12	2.55	**.01**
Females per plant	−0.37 ± 0.3	−1.23	.22
Males per plant	0.21 ± 0.29	0.74	.46
Mating success	0.1 ± 0.11	0.85	.39
Females per plant × spatial centrality	0.53 ± 0.28	1.91	.06
Males per plant × spatial centrality	−0.34 ± 0.28	−1.19	.23
Mating success × spatial centrality	0.02 ± 0.1	0.18	.85
*Males*			
(Intercept)	−0.26 ± 0.12	−2.16	**.03**
Spatial centrality	0.22 ± 0.11	1.91	.06
Females per plant	−0.71 ± 0.23	−3.05	**<.01**
Males per plant	0.51 ± 0.23	2.19	**.03**
Mating success	0.09 ± 0.1	0.87	.38
Females per plant × spatial centrality	0.48 ± 0.25	1.95	.051
Males per plant × spatial centrality	−0.37 ± 0.25	−1.51	.13
Mating success × spatial centrality	−0.08 ± 0.1	−0.78	.44

Model coefficients are presented as estimate ± standard error, and the x denotes statistical interactions between two variables. All continuous predictor variables were centered and standardized prior to model fitting, so that coefficients are comparable between different models.

### 3.2 Where to go?

Here, we analyzed 495 plant switches performed by 215 males and 430 switches performed by 163 females. Male movement was influenced by both spatial distance between plants and female availability: Males moved preferentially to spatially close plants and to plants with more females. The number of males per plant, however, did not influence male movement (Table 2). Females were also more likely to move between spatially close plants and responded to both the number of males and females per plant. They moved preferentially toward plants with more females and fewer males (Table 2).

**Table 2:** Summary of the multinomial network models used to investigate *where* males and females of the leaf beetle *Leptinotarsa undecimlineata* should go after leaving a host plant

Predictor variable	Parameter symbol	Median estimate	95% Credible interval	MCMC *p*‐value
*Females*				
Spatial distance	*A*	−5.27	−6.19 to −4.49	<.001
Males per plant	*B*	−0.15	−0.31 to −0.005	.02
Females per plant	*C*	0.24	0.09 to 0.40	<.001
(Individual random effect variation)	*A*_σ_	2.78	2.17–3.55	‐
(Host plant random effect variation)	*G*_σ_	0.97	0.79 to 1.119	‐
*Males*				
Spatial distance	*A*	−3.97	−4.66 to −3.38	<.001
Males per plant	*B*	−0.07	−0.20 to 0.05	.131
Females per plant	*C*	0.14	0.01 to 0.27	.014
(Individual random effect variation)	*A*_σ_	2.35	1.85 to 2.95	‐
(Host plant random effect variation)	*G*_σ_	1.13	0.94 to 1.37	‐

Parameter symbols are those used in the model's equations (see topic *WHERE TO GO?* in the Section 2). Random effect variation is presented as standard deviation. All predictor variables were centered and standardized prior to model fitting, so that coefficients are comparable between different models.

## 4 DISCUSSION

Here, we studied the movement patterns of males and females of a leaf beetle to test the general hypothesis that individual movements in scramble competition polygynies (SCPs) are driven by the search for mating opportunities. We separated mate searching into two main decisions of the individuals: *when to move* and *where to go*. Regarding the *when to move* decision, we found that males leave their sites when they encounter few females and many males. Females, in turn, are more likely to move when they are in centrally located plants, but do not leave their sites in response to the social environment, that is, the number of potential mates and competitors. Regarding the *where to go* decision, both males and females were more likely to move toward plants within short distances. This may be a way of avoiding long movements between sites, which may be energetically costly and may expose individuals to predators. Furthermore, short movements make it easier for an individual to return to their original site if the new site turns out to be unfavorable. Our results also show that females are more reluctant than males to switch between more distant host plants, which may be a consequence of higher mobility costs for females when they are bearing eggs (see Figure 1). Finally, whereas males are more likely to move to plants with more females, females prefer plants with more females and less males.

Contrary to our initial prediction, female behavior was more consistent with a harassment avoidance strategy than with a mate searching strategy because they avoided plants with many males. Although females of closely related chrysomelid species benefit from multiple mating, there is an optimum number of copulations above which mating costs do not pay off (Fan, Wang, Li, & Zhang, 2014). Excess copulations may impose direct costs to females (den Hollander & Gwynne, 2009; Maklakov & Lubin, 2004), and male harassment per se has been reported to impose costs to females of many species (Magurran & Seghers, 1994; Gay et al.^2009^, Chang & Sih, 2013). These costs may explain why avoiding places with many males is a relatively frequent behavior in females of vertebrates and invertebrates (Odendaal, Turchin, & Stermitz, 1989; Wearmouth et al., 2012). Male avoidance behavior may also explain the negative relationship between movement and mating rate previously reported for females of *L. undecimlineata* (Baena & Macías‐Ordóñez, 2015). If females are actively avoiding plants with many males, more mobile females would encounter fewer males and therefore copulate less frequently. We propose that, after reaching the optimum number of copulations and securing fertilization, females start avoiding male harassment and searching for adequate oviposition sites. The search for adequate oviposition sites may also explain why females are seeking plants with other females: They are probably responding to the same cues of host plant quality, or even using the number of females as a proxy of plant quality. In addition, females may prefer sites with many other females to dilute the risk of male harassment among more females, or the risk of egg predation. Based on our results, we predict that females suffering costs of male harassment and living in dense populations should seek sites or regions with few males and many females, as a strategy that would decrease the risk of male harassment and perhaps increase offspring protection.

In SCPs, sexual selection is expected to favor male locomotor ability (reviewed in Herberstein et al., 2017). Studies that reported positive relationships between movement and mating frequency usually focused on species in which females are highly philopatric and sometimes show aggressive behavior against other females (Lane, Boutin, Gunn, & Coltman, 2009; Sandell, 1986; Stockley et al., 1994). In these cases, males need to move to find receptive females, and once they have found a female and either have been accepted or rejected, they must move to find another mate. The only two cases of a negative relationship between movement and mating frequency for males were reported for the leaf beetle *L. undecimlineata* (Baena & Macías‐Ordóñez, 2015) and a population of the water snake *Nerodia sipedon* (Brown & Weatherhead, 1999). In both cases, population density was very high and, after finding a female, a male had the potential to find additional females with little or no movement. Although males of *L. undecimlineata* did not base their decision of *when* to move on their own mating success, they were more likely to leave sites when the local number of females was low and the number of males was high. We interpret this result as a strategy that promotes further copulation opportunities and avoids local competition with other males. This strategy may only be profitable when the mating sites are spatially patchy (e.g., pools and host plants) and the density of potential mates (females) is high. When mating sites are spatially scattered and the density of potential mates is low, males should heavily invest in mate searching and a positive relationship between movement and mating frequency should be expected.

Males deciding *where* to go did not respond to the number of males in the new plant, despite the fact that they have higher probability of leaving plants when they contain many males. The effect of the number of males, however, was much weaker than the effect of the number of females when males were deciding when to leave a plant (Table 1). Overall, this result indicates that males are responding more strongly to mating opportunities than to the presence of potential competitors. The weak response to mate competition may occur because females are highly polyandrous and aggressive interactions between males are rare in *L. undecimlineata* (Baena & Macías‐Ordóñez, 2012). As expected, males preferred to move toward plants with more females. This movement pattern could be a response to airborne pheromones released by receptive females. However, because closely related chrysomelid species cannot distinguish male and female conspecifics from a distance (Nahrung & Allen, 2004), other mechanisms may be involved. We suggest that both males and females may simply switch to neighboring plants and decide whether to stay or to promptly switch again (within minutes or a few hours) depending on local conditions, such as the number of conspecific males and females. Thus, our daily records would reflect the “final” decision of each individual after sampling one or more sites. Regardless of the mechanism, males seem to be moving in search of mating opportunities, so that males with low encounter rate with females are more likely to move. This may explain the previously reported negative correlation between male movement and mating success in *L. undecimlineata* (Baena & Macías‐Ordóñez, 2015).

Despite some differences to the expected patterns, male movements were consistent with the following rules: leave the site when mating opportunities are low, when there are many competitors, and seek nearby sites where mating opportunities are high. These rules may reduce the mate searching costs and maximize reproductive success, representing an optimal strategy that we expect to be present in other species with a SCP mating system in which mating sites are distributed in discrete patches. The concept of optimization has long permeated the literature on foraging behavior because the net benefit of feeding incorporates both costs associated with finding food and benefits derived from food quality and quantity (Schoener, 1971). We argue that the same rationale could also be applied to generate predictions regarding mate searching. If we assume that mate searching is costly and potentially risky (Byers, Wiseman, Jones, & Roffe, 2005; Lane, Boutin, Speakman, & Humphries, 2010; Polis et al., 1998), and that reproductive success increases with the number of copulations (Bergeron, Montiglio, Réale, Humphries, & Garant, 2012; Fritzsche & Arnqvist, 2013), sexual selection should favor strategic movements that minimize searching costs and risks and maximize mating opportunities. This is especially true for males, but also applies to females that benefit from multiple copulations. Furthermore, under such a scenario, both males and females are expected to avoid high male density, avoiding competition and harassment, respectively. Thus, predictions regarding strategic mate search should take into account the ecological and social context experienced by each individual.

Andersson (1994) describes *scramble* as a mechanism of mate competition in which males compete to be the first to find a female and defines SCPs as mating systems in which scrambling is the main mechanism of mate competition. As noted by Herberstein et al. (2017), this definition of SCP includes at least two mating systems: (1) explosive breeding, in which there is little or no time for male‐male contest or female choice before the end of the breeding season, and (2) prolonged searching polygyny, in which the breeding season is long and there is more potential for mate competition and female choice. Considering that *scramble competition* is a mechanism of mate competition, not a mating system per se, this mechanism may be present as an additional competition mechanism in any mating system (Baena & Macías‐Ordóñez, 2015). In resource defense polygynies, for instance, males compete for females via territorial contests, but frequently there are sneaker males that move between territories and invade them to copulate furtively (Oliveira, Taborsky, & Brockman, 2008). Thus, sneaker males compete via scrambling because their mating success depends on finding territories and females. Similarly, males seeking extra‐pair copulations in monogamous species compete via scrambling because they must find receptive paired females to copulate with (Westneat & Stewart, 2003). Therefore, we expect to find strategic mate searching movements not only in SCPs, but also in any mating system in which individuals compete for mates, at least in part, via scrambling. This is a new way of looking at mate searching that provides testable hypotheses, such as the ones we tested here, and that can generate useful insights into the study of individual movements in natural populations.

## CONFLICT OF INTEREST

None declared.

## AUTHORS' CONTRIBUTIONS

MLB and RM collected the data. DGM performed the statistical analyses. All authors participated in the elaboration of the hypotheses, discussion of the results, and writing of the manuscript.

## DATA ACCESSIBILITY

Should the manuscript be accepted, the full dataset used in this manuscript will be available at Zenodo (https://zenodo.org/).
